# Evaluation of symptoms in respiratory syncytial virus infection in adults: psychometric evaluation of the Respiratory Infection Intensity and Impact Questionnaire™ symptom scores

**DOI:** 10.1186/s41687-023-00593-9

**Published:** 2023-06-01

**Authors:** Richard H. Osborne, Lauren M. Nelson, Sheri Fehnel, Nicole Williams, Randall H. Bender, Ryan Ziemiecki, Efi Gymnopoulou, Els De Paepe, Yannick Vandendijck, Lindsey Norcross, Esther Heijnen, Gabriela Ispas, Christy Comeaux, Benoit Callendret, Eric K. H. Chan, Jane A. Scott

**Affiliations:** 1 Measured Solutions for Health, P.O. Box 5127, Alphington, VIC 3079 Australia; 2 Centre of Global Health and Equity, Swinburne University of Technology, Hawthorn, VIC Australia; 3 RTI Health Solutions, Research Triangle Park, NC USA; 4 Janssen Infectious Diseases, Beerse, Antwerp, Belgium; 5 Janssen Global Services, Raritan, NJ USA; 6 Janssen Global Services, High Wycombe, Buckinghamshire, UK

**Keywords:** RiiQ™, Respiratory syncytial virus, Validation, Psychometric, Patient-reported outcome

## Abstract

**Background:**

The Respiratory Infection Intensity and Impact Questionnaire (RiiQ™) is a patient-reported outcome measure designed to assess symptoms and impacts of respiratory syncytial virus (RSV) infection. This study evaluated the construct validity, reliability, and responsiveness of the RiiQ™ Respiratory and Systemic Symptoms Scale scores.

**Methods:**

Prospective data were analyzed from a total of 1795 participants, including from non-hospitalized patients with acute respiratory infection (ARI) and no coinfections enrolled in a Phase 2b RSV vaccine study (RSV-positive: n = 60; RSV-negative: n = 1615), and two observational studies of patients hospitalized with RSV (n = 20; n = 100). Descriptive statistics, confirmatory factor analysis (CFA), test–retest intraclass correlation coefficients (ICCs), construct validity correlations (between a clinician-assessed clinical questionnaire and the RiiQ™ symptoms scale), known-groups validity, and responsiveness (correlations of change scores) were evaluated.

**Results:**

Mean patient age ranged from 66.5 to 71.5 years and the majority of patients were female. Initial assessments in the vaccine trial (ARI Day 1) were suggestive of less severe illness than in the observational studies with hospitalized patients. CFA loadings (> 0.40) supported summary scores. ICCs exceeding the recommended threshold of 0.70 supported test–retest reliability for Respiratory and Systemic Symptoms, except in the small observational study. At the scale level, correlations were moderate to strong (|*r*| ≥ 0.3) and positive between the Respiratory Symptoms Scale and the related clinical questionnaire scores, reflecting measurement of similar symptoms in support of convergent validity. Correlations with change in Patient Global Impression of Severity > 0.30 supported responsiveness.

**Conclusions:**

Psychometric tests applied to the RiiQ™ Symptoms scales provide evidence of its reliability, construct validity, discriminating ability, and responsiveness for use in clinical studies to assess the onset and severity of RSV symptoms.

**Supplementary Information:**

The online version contains supplementary material available at 10.1186/s41687-023-00593-9.

## Background

Respiratory syncytial virus (RSV) is a common virus that can present a serious health concern for high-risk populations, including young children, adults aged ≥ 60 years, and individuals with chronic heart or lung disease or with compromised immune function [1]. RSV infection, while often underdiagnosed in older adults, represents a major cause of serious respiratory illness and increases mortality risk in this population [2, 3]. It has been estimated that 11,000 people aged ≥ 65 years die annually in the United States (US) as a result of RSV [2].

Symptoms of RSV, including cough, fever, nasal congestion, wheezing, shortness of breath, sore throat, headache, and myalgia, negatively impact health-related quality of life—particularly among older and other high-risk individuals, who may experience more severe respiratory illness [4]. Symptoms of RSV are diverse and can be challenging to measure reliably, as they may appear, subside, or resolve at different times and rates. To effectively assess the impact of prophylactic and/or therapeutic agents, a patient-reported outcome (PRO) measure that is both able to detect the onset of RSV symptoms and sensitive to changes in symptom severity is needed [5, 6]. The Respiratory Infection Intensity and Impact Questionnaire (RiiQ™), a PRO measure of symptom severity and impacts of RSV infection, was developed for use in clinical trials to evaluate the efficacy of new RSV vaccines and treatments. Consistent with Food and Drug Administration guidance [5], the RiiQ™ was adapted from a well-developed PRO, the Influenza Intensity and Impact Questionnaire (FluiiQ™), a measure widely used to detect and monitor symptoms of influenza and acute respiratory infection (ARI) [6, 7].

The current study involved conducting a psychometric evaluation of the RiiQ™ in accordance with regulatory guidance and best practice recommendations [5, 8, 9]. Specifically, data collected from a vaccine trial and from two observational studies designed to monitor disease course and symptoms in adults with RSV were used to evaluate the measurement properties of the RiiQ™ symptom scales.

## Methods

### Development and overview of the RiiQ™

The RiiQ™ was developed based on the theoretical structure and content of the FluiiQ™ [7]. After initial adaptation of the RiiQ™, a qualitative interview study was conducted to iteratively refine the questionnaire [6]. This research established content validity, as study participants deemed the concepts included in the measure both relevant and important and found the instructions, questions, and response options easy to understand and complete.

**Fig. 1:**
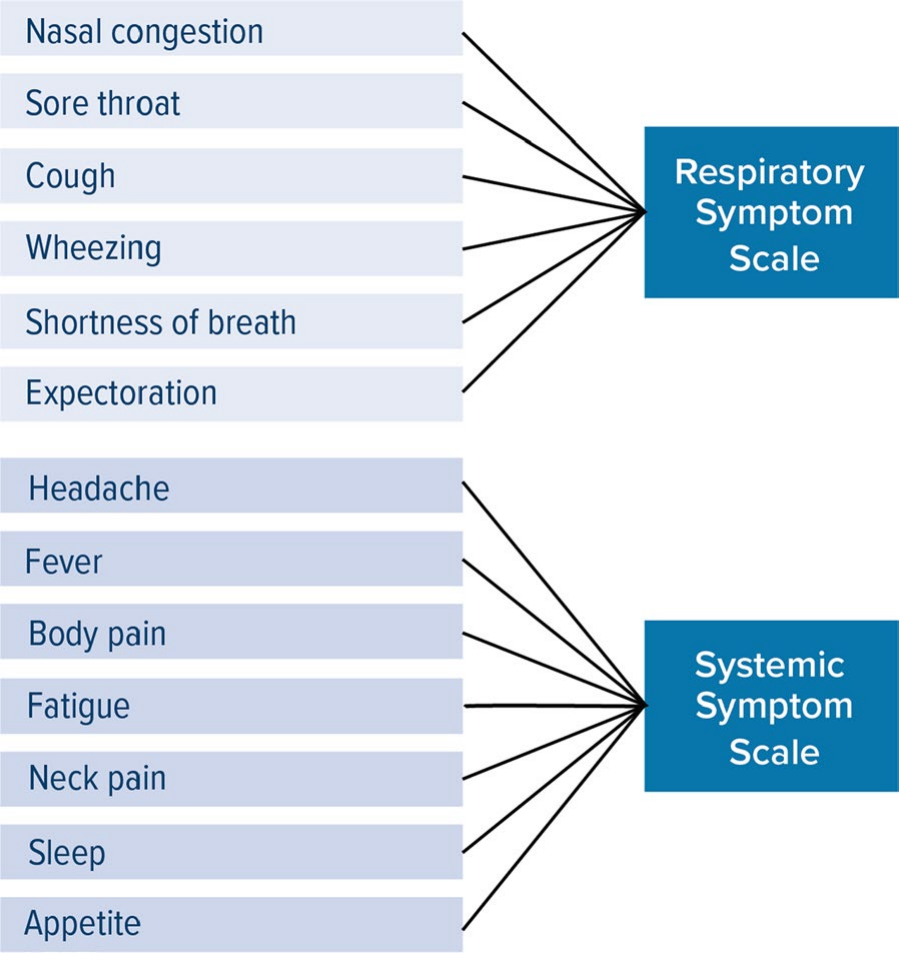
RiiQ™ Symptoms Scale. *RiiQ™* Respiratory Infection Intensity and Impact Questionnaire

The RiiQ™ comprises four sections. “Background” section, which covers RSV symptoms, asks respondents to indicate the presence and severity of six symptoms related to the upper respiratory tract (URT) and lower respiratory tract (LRT) (cough, sore throat, nasal congestion, wheezing, expectoration, shortness of breath) and seven symptoms related to systemic responses to the infection (headache, fever, body pain, fatigue, neck pain, sleep, and appetite) (Fig. 1). Response options include 0 = None, 1 = Mild, 2 = Moderate, and 3 = Severe, and recall period is the past 24 h (to facilitate daily administration of the measure). The remaining three sections of the RiiQ™ are not explored in this paper and include: 2. Impact on daily activities, 3. Impact on emotions, and 4. Impact on other people. The RiiQ™ was used under license from Measured Solutions for Health P/L.

### Data sources

Prospective data from three different studies—a vaccine trial and two observational studies—were used to evaluate the psychometric properties of the RiiQ™ symptoms scales. Patient characteristics and disease status (RSV-positive/negative) were collected in each study. Additional file 1: Tables S1 and S2 present the assessment schedule for the RiiQ™ and an overview of other measures administered in each study.

VAC18193RSV2001, a phase 2 vaccine trial (NCT03982199), was conducted to evaluate the efficacy of an RSV vaccine during the RSV season after a single vaccination [10]. A total of 5782 US participants were enrolled and randomized (in equal numbers) to receive active study vaccine or placebo. Participants completed the RiiQ™ on an eDevice at baseline (before vaccination), at the visit at the end of the RSV season, at home each day during an ARI episode, and at prespecified site visits during an ARI episode (e.g., at site visits including ARI Day 3–5 and Day 29). In addition to the RiiQ™, participants completed other PRO measures including the Lawton–Brody Instrumental Activities of Daily Living (IADL) scale, the Patient Global Impression of Severity, and the Patient Global Impression of Health; a clinical questionnaire was also administered. An ARI episode commenced when a participant reported experiencing at least one respiratory symptom that was new or worse than usually experienced at baseline. Resolution of an ARI episode was defined as two consecutive days with no symptoms listed on the RiiQ™ Symptoms Scale, or for participants who had symptoms on the RiiQ™ Symptoms Scale present at baseline, as two consecutive days on which all symptoms on the RiiQ™ Symptoms Scale returned to the severity reported at baseline. Participants who experienced symptoms suggesting an ARI episode were tested for RSV via an RT-PCR assay. Data from the primary analysis (i.e., through the end of the first RSV season) were used in this evaluation.

NOPRODRSV0006 (Observational Study 1; HARTI) was a longitudinal study of adults hospitalized with ARI during the influenza/RSV/human metapneumovirus (hMPV) season at multiple centers located in 12 different countries around the world (northern and southern hemisphere) [11]. This analysis used an interim dataset that included patients enrolled before 1 January 2019 and with a hospital stay of more than 48 h. Approximately 3600 patients were enrolled in the main study, which was limited to one day (i.e., within 24 h of hospitalization), during which screening and initial assessments, including diagnostic testing, were performed. Of those with confirmed influenza, RSV, or hMPV infection, 532 were enrolled in a substudy, wherein the RiiQ™ and other outcome measures (clinical questionnaire, Lawton–Brody IADL, Respiratory Symptoms Bother, and Change in Health Status) were administered. The substudy consisted of two consecutive phases: (1) a hospitalization phase with up to three timepoints (48 h after the screening/initial assessment visit, within approximately two days before the planned hospital discharge, and at hospital discharge); and (2) a follow-up phase with phone interviews at one, two, and three months after discharge.

NOPRODRSV0004 (Observational Study 2) was a longitudinal study that enrolled 24 hospitalized adults presenting with ARI during the RSV season in Belgium. A total of 20 RSV-positive patients were enrolled and completed the study. Patients enrolled at screening and with confirmed RSV infection entered a hospitalization phase with daily visits (for up to seven days and, in case of hospitalization longer than seven days, a before-discharge visit a maximum of two days before hospital discharge). A follow-up phase after discharge consisted of phone interviews in 10-day intervals for up to 30 days (until the patient reported returning to their usual health state). The Respiratory Symptoms Bother and Change in Health Status scales and the RiiQ™ Symptoms Scale were completed daily from Day 2 to Day 7 of the hospitalization phase (at most two days before discharge and at the before-discharge visit) and remotely through a phone call at Day 10 (± 2 days) after hospital discharge. If the patient still reported respiratory symptoms on the RiiQ™ Symptoms Scale, Respiratory Symptoms Bother, and Change in Health Status, and/or the answer to the question “Have you returned to your usual health” was “No,” the remote monitoring phase was extended until these symptoms were resolved, with a maximum of two additional phone calls (at 20 ± 2 days after hospital discharge and at 30 ± 2 days after hospital discharge).

### Other measures used in the evaluation

The clinical questionnaire, Return to Usual Health item, and Lawton–Brody IADL scale were completed in all three studies. The three global impression items were completed daily during an ARI episode in the vaccine trial. The Responsiveness item was completed only in the observational studies.

### Clinical questionnaire

The severity of 11 respiratory symptoms was rated from 0 (No symptoms) to 3 (Bothersome most of the time, interfering with other activities) by clinic staff (with patient input). A total Clinical Symptom Score was computed as the sum of the 11 symptom scores, ranging from 0 to 33 points, with higher scores indicating more severe RSV-related illness. The 11 symptoms were further divided into categories: general respiratory symptoms (cough, sputum production, shortness of breath, and malaise), URT symptoms (nasal discharge, pharyngitis, and sinus tenderness), and LRT symptoms (dyspnea; rales, rhonchi, or other; wheezing; and respiratory effort). The respiratory effort item was not administered in the HARTI study.

The clinical questionnaire was completed during the vaccine trial at the beginning of the study and at each site visit during an ARI period (ARI Day 3–5 and Day 29). In HARTI, the clinical questionnaire was completed at screening, at 48 h after the screening visit, and within approximately two days before planned hospital discharge. In Observational Study 2, the clinical questionnaire was completed at screening (Day 1), daily during the assessment phase (Day 2 to Day 7), and before hospital discharge (at most two days before discharge).

### Lawton–Brody IADL scale

Participants were asked to rate their functioning in eight domains required for independent living, including ability to use a phone, shopping, food preparation, housekeeping, laundry, mode of transportation, responsibility for own medications, and ability to handle finances [12]. Scoring was 0 (Low Function/Dependent) to 8 (High Function/Independent).

### Patient global impression of severity (PGI-S)

The PGI-S asked participants to rate the severity of their respiratory illness on a four-point scale from 0 (I feel fine) to 3 (I feel extremely ill).

### Patient global impression of health (PGI-H)

The PGI-H asked participants to rate their overall health status on a five-point scale from 0 (Very poor) to 4 (Very good).

### Patient global impression of change (PGI-C)

This PGI-C asked participants to rate any change in their health on a seven-point scale ranging from –3 (Much better) to 3 (Much worse).

### Responsiveness item

Participants were asked to rate their perception of change: “Since this time yesterday, are you…” (for an in-hospital interview) or “Since last interview, are you…” (for a remote interview). Response categories included Much worse, Worse, The same, Better, or Much better.

### Psychometric analyses

Analyses to evaluate measurement properties for the RiiQ™ Respiratory and Systemic Symptoms Scales were conducted using data from the vaccine trial and HARTI. Given the small sample size (n = 24), data from Observational Study 2 were used only to augment the descriptive statistics, for construct validity, and test–retest reliability analyses. The psychometric assessments included descriptive characteristics, structure (scoring), test–retest reliability, construct validity, discriminating ability, and ability to detect change. Descriptions of the analysis methods follow.

Data from the initial ARI assessment in HARTI (i.e., baseline; n = 100) and in the vaccine trial (i.e., ARI Day 1; n = 60) were used to evaluate structure and scoring, given appropriate sample sizes. Specifically, inter-item and item-to-total correlations, as well as Cronbach’s alphas [13], were computed using both sets of data to evaluate internal consistency (reliability), and a series of confirmatory factor analysis (CFA) models were fitted to the HARTI data. The underlying relationships of the RiiQ™ items were evaluated via a series of effect-indicator CFA models (using Mplus version 7.4) and via weighted least squares mean and variance-adjusted estimation. Following previous efforts for model fitting the FluiiQ™ scale [7], effect-indicator or reflective models were fitted for the RiiQ™, allowing for the underlying assumption that the symptoms are considered highly related and positively intercorrelated. The criteria for CFA model fit included the commonly used chi-square comparative fit index (CFI), the Tucker–Lewis Index (TLI) [14], and the root mean square error of approximation (RMSEA [15, 16]). For both the CFI and TLI fit indices, values > 0.95 are most desirable for controlling type I and type II error rates; values ≥ 0.90 to 0.95 are considered marginal; and values < 0.90 indicate poor model fit. The RMSEA was evaluated such that values < 0.06 suggested satisfactory model fit, ≥ 0.06 to < 0.08 fair model fit, ≥ 0.08 to 0.10 mediocre model fit, and > 0.10 poor model fit [15]. Good model fit with generally high loadings (≥ 0.5) confirms domain scoring of each domain in the model and, combined with a strong inter-factor correlation, confirms use of a total score.

The test–retest reliability of the RiiQ™ scales was assessed by computing intraclass correlation coefficients (ICCs) for participants who were RSV-positive, whose disease severity was considered stable for two consecutive days, and who had no change as assessed by either the Responsiveness item or the PGI-S measures. ICC values were evaluated according to recommended guidelines of ≥ 0.70 for multiple item measurement scales [17]. A two-way mixed-effects analysis of variance (ANOVA) model with absolute agreement for single measures was used to compute the ICCs between scores from the two timepoints [18–20].

To assess construct validity, or the degree of relatedness or dissimilarity between the RiiQ™ Symptoms Scale scores and the comparison measures (clinical questionnaire, PGI-H, PGI-S, and Lawton–Brody IADL scores), correlations were computed and evaluated based on Cohen’s guidelines [13]. Pearson correlations were computed between the RiiQ™ Symptoms Scale summary scores and the related clinical questionnaire and Lawton–Brody IADL summary scores, and polyserial correlations were computed between the global measures and the Lawton–Brody IADL item scores.

In known-groups analyses, the discriminating ability of the RiiQ™ Respiratory and Systemic Symptoms Scale scores was compared between groups formed from comorbid conditions of asthma or chronic obstructive pulmonary disease (COPD) versus groups with no asthma or COPD, as well as between groups derived from the PGI-S and the PGI-H. Higher (worse) RiiQ™ Respiratory and Systemic Symptoms Scale scores were anticipated for groups with asthma/COPD, with more severe respiratory illness (as indicated by the PGI-S or PGI-H), and with worse health status (as indicated by the PGI-H). ANOVAs were conducted for the entire ARI sample (with no coinfections) from the vaccine trial under the assumption that the RiiQ™ summary scores would behave similarly regardless of infection status (an ANOVA test of this assumption was not significant, *P* > 0.05). Data collected on ARI Day 1 were used for the PGI-S and PGI-H comparisons. Finally, descriptive analyses were conducted and Pearson correlations computed with each of the global items to evaluate the ability of the RiiQ™ Symptoms Scale to detect responsiveness to change using the entire ARI vaccine trial sample (with no coinfections).

## Results

### Participant characteristics

In the RSV-only subgroups of the three studies, there were more females than males (61.7% from the vaccine trial, 62.0% from HARTI, and 58.3% from Observational Study 2), the mean (SD) age ranged from 66.5 (17.9) to 71.5 (5.1) years, and the mean (SD) number of comorbidities ranged from 1.5 (1.1) to 2.7 (2.0) (Additional file 1: Table S3). While the vaccine trial was conducted in the US and the sample was predominantly White (100.0%) and non-Hispanic (100.0%), the observational studies were conducted globally.

### Clinical characteristics of the study sample

Although symptoms such as fever, body pain, neck pain, and loss of appetite were commonly absent or reported as mild at the initial assessment across all three studies (Additional file 1: Table S4), other symptoms such as cough and fatigue were more often reported as moderate to severe (Additional file 1: Table S5). Consistent with the context of the three studies, initial assessments in the vaccine trial (ARI Day 1) were suggestive of less severe illness than in the observational studies with hospitalized patients.

Follow-up assessments suggested that RSV-related illness had resolved within the 29-day period for most vaccine study participants, while symptom resolution was less marked in the observational studies involving hospitalized patients. Mean LRT symptom summary scores were higher than mean URT symptom summary scores in HARTI (hospital setting), while mean LRT symptom summary scores were lower than mean URT summary scores in the vaccine study (Additional file 1: Table S6). The lowest scores (i.e., no symptoms) were reported most commonly for URT symptoms in HARTI (hospital setting) (28.0%) and for LRT symptoms in the vaccine study (7.5%).

### Psychometric properties

#### Structure and scoring

For the Respiratory Symptoms Scale, inter-item correlations between the LRT symptoms cough, wheezing, expectoration, and shortness of breath were consistently moderate (*r* > 0.36) across the two studies, except for the small correlation (*r* = 0.21) observed between expectoration and shortness of breath in HARTI (Table 1). However, the correlations between the URT symptoms sore throat and nasal congestion (− 0.16 [vaccine trial] and 0.35 [HARTI]) and the pattern of correlations between the systemic symptoms were inconsistent between the two studies.

**Table 1:** Respiratory symptoms inter-Item (Polychoric) correlations in participants with RSV-positive disease status

RiiQ™ respiratory item^†^	Polychoric correlation				
	a. Cough	b. Sore throat	d. Nasal congestion	j. Wheezing	k. Expectoration
Vaccine trial—ARI PRO eDiary Day 1 (n = 40)					
a. Cough	–				
b. Sore throat	0.37 (0.04, 0.69)	–			
d. Nasal congestion	− 0.03 (− 0.40, 0.33)	− 0.16 (− 0.53, 0.20)	–		
j. Wheezing	**0.53** **(0.22, 0.84)**	0.27 (− 0.11, 0.65)	− 0.10 (− 0.51, 0.31)	–	
k. Expectoration	**0.58** **(0.32, 0.84)**	0.19 (− 0.17, 0.54)	0.02 (− 0.35, 0.40)	**0.55** **(0.26, 0.85)**	**–**
l. Shortness of breath	**0.69** **(0.44, 0.94)**	0.29 (− 0.09, 0.68)	0.27 (− 0.12, 0.67)	**0.53** **(0.19, 0.87)**	**0.84** **(0.69, 1.00)**
HARTI—baseline (n = 100)					
a. Cough	–				
b. Sore throat	0.53 (0.31, 0.74)	–			
d. Nasal congestion	0.33 (0.13, 0.54)	0.35 (0.10, 0.60)	–		
j. Wheezing	**0.62** **(0.47, 0.78)**	0.39 (0.15, 0.63)	0.47 (0.29, 0.66)	–	
k. Expectoration	**0.36** **(0.16, 0.56)**	0.23 (− 0.03, 0.49)	0.24 (0.03, 0.46)	**0.37** **(0.17, 0.57)**	**–**
l. Shortness of breath	**0.65** **(0.51, 0.80)**	0.51 (0.29, 0.72)	0.40 (0.20, 0.60)	**0.76** **(0.65, 0.87)**	**0.21** **(− 0.01, 0.43)**

Bolded cells indicate a lower respiratory tract symptom

*ARI* acute respiratory infection, *PRO* patient-reported outcome, *RiiQ™* Respiratory Infection Intensity and Impact Questionnaire, *RSV* respiratory syncytial virus

^†^Items truncated. Complete items available from the author

**Fig. 2:**
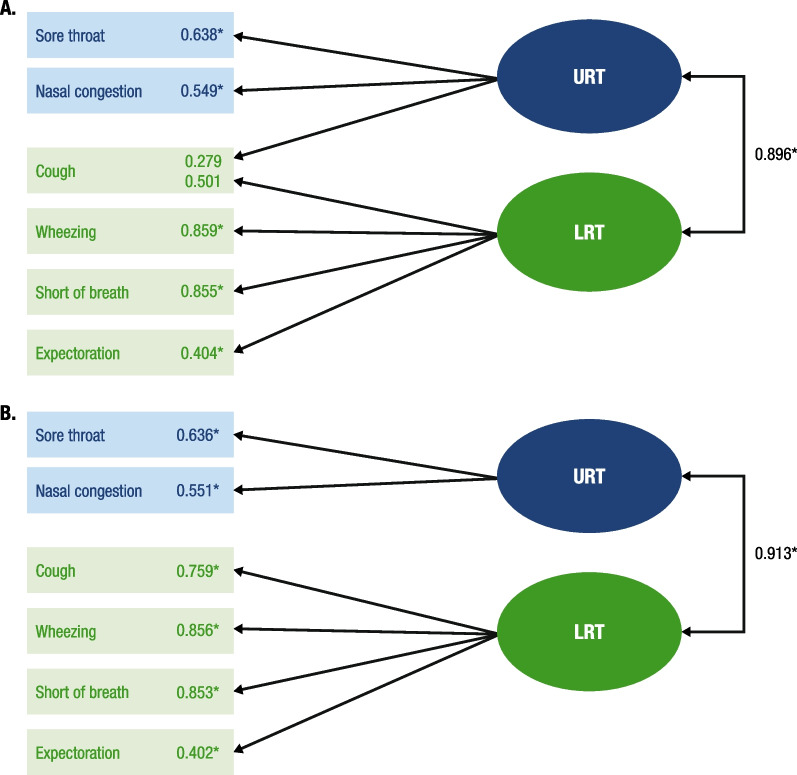
Respiratory System Scale CFA loadings^a^ at baseline in sample of hospitalized patients: HARTI (N = 100). **A** Cough loaded on LRT and URT. **B** Cough loaded on LRT only. *CFA* confirmatory factor analysis, *CFI* comparative fit index, *CI* confidence interval, *LRT* lower respiratory tract, *RMSEA* root mean square error of approximation, *RSV* respiratory syncytial virus, *TLI* Tucker–Lewis index, *URT* upper respiratory tract, *WRMR* weighted root mean square residual. ^a^Factor loadings and covariance are standardized and thus represent correlations. *P < 0.05. Sample: RSV-positive, observational study at baseline. Model fit statistics for cough on URT and LRT: CFI = 0.999; TLI = 0.997; RMSEA (95% CI) = 0.025 (0–0.127); chi-square test value (*df* = 7) = 7.429 (*P* = 0.3857); WRMR = 0.340. Model fit statistics for cough on LRT only: CFI = 1.000; TLI = 1.000 (estimate > 1.000 set to 1.000); RMSEA (95% CI) = 0.000 (0–0.115); chi-square test value (*df* = 8) = 7.728 (*P* = 0.4604); WRMR = 0.344

For data collected in HARTI from the Respiratory Symptoms Scale, two different two-factor CFA models were fitted so that the cough symptom was loaded on both the LRT and URT factors in the first model and loaded only on the LRT factor in the second model (Fig. 2). This exploration was performed because coughing can arise from either local irritation in the throat (ie, URT) or from mucus production lower in the respiratory system (ie, LRT). The fit was good in both models, and the factor loadings were all at least 0.40, except for cough, which had a loading of 0.28 when reflecting only the URT factor. Thus, results suggest that the simpler (second) model with cough loading only on the LRT was the better solution. Further, a chi-square difference test yielded no significant difference between the two models.

#### Reliability

In the analyses of internal consistency, Cronbach’s alpha values were within the optimal range (0.70–0.90) [21] at all timepoints, except for two cases using data from the 40 to 53 RSV-positive study participants in the vaccine trial (Table 2). However, these values were close to the lower bound of this range at 0.68 (Respiratory) and 0.61 (Systemic).

**Table 2:** RiiQ™ Symptoms Scale internal consistency reliability in participants with RSV-positive disease status

Study/time period	Cronbach’s alpha, n	
	Respiratory symptoms	Systemic symptoms
Vaccine trial		
ARI Day 1 (PRO eDiary)	0.68, 40	0.82, 40
ARI Day 3–5 (site visit)	0.74, 43	0.78, 41
ARI Day 29 (site visit)	0.72, 53	0.61, 53
HARTI		
Baseline^†^	0.76, 100	0.73, 100
Prior to discharge	0.73, 54	0.79, 54
Follow-up	0.70, 86	0.71, 86

*ARI* acute respiratory infection, *RiiQ™* Respiratory Infection Intensity and Impact Questionnaire, *RSV* respiratory syncytial virus

^†^Initial (baseline) assessment was a maximum of 48 h after the screening visit

The ICCs exceeded the recommended threshold of 0.70 [17] for LRT, URT, Respiratory Symptoms, and Systemic Symptoms, except in the small observational study (Observational Study 2), indicating good test–retest reliability for these scores (Additional file 1: Table S7). Notably, estimates based on vaccine trial data had tighter 95% confidence interval bands, possibly because of the larger sample size and the use of the PGI-S to define stability (rather than the Responsiveness item, as in Observational Study 2).

#### Construct validity

Correlations between the clinical questionnaire and RiiQ™ Symptoms Scale item-level scores demonstrated strong concordance between clinician and patient self-ratings of cough, shortness of breath, and wheezing symptoms (see Additional file 1: Table S8); they demonstrated inconsistency for the other symptoms, possibly due to lack of variability in the scores or overlap in the timing of the assessment. At the scale level, correlations were moderate to strong (|*r*| ≥ 0.3) and positive between the Respiratory Symptoms Scale and the related clinical questionnaire scores, reflecting measurement of similar symptoms in support of convergent validity (Additional file 1: Table S9). There were a few instances of smaller-than-anticipated correlations between the URT and the clinical questionnaire upper respiratory scores using data from the smaller samples. Overall, there was a clear pattern where similar concepts were more highly correlated than less similar concepts.

Polyserial correlations were moderate to strong (|*r*| ≥ 0.3) between the Respiratory Symptoms Scale scores and the PGI-S (positive; higher scores indicate a worse condition) and PGI-H (negative; higher scores indicate a better condition) scores, supporting convergent validity, with a few exceptions with the URT and Systemic score correlations (Additional file 1: Table S10). The Pearson correlations were trivial to small (|*r*| < 0.3) between the Respiratory Symptoms Scale scores and Lawton–Brody IADL total score, except for the nonsignificant moderate to strong correlations computed with data in the small observational study (Additional file 1: Table S11).

#### Discriminating ability

In the known-groups analyses, observed trends support the expectations for the asthma/COPD comparison, where mean Respiratory and Systemic Symptoms scores in the group with asthma/COPD tended to be higher than scores in the group without asthma or COPD (Additional file 1: Table S12). A similar pattern of increasing symptoms scores with increasing severity as indicated by the PGI-S was also observed (Additional file 1: Table S13). Results for the PGI-H also met expectations (Additional file 1: Figure S1).

#### Ability to detect change

The ability of the RiiQ™ to detect change was confirmed by a review of the patterns of change in RiiQ™ Symptoms Scale scores across the change in PGI-S subgroups and the change in PGI-H subgroups (Additional file 1: Table S14), and less so across the PGI-C levels. Among participants with changes in global item scores reflecting improvement, the RiiQ™ Symptoms Scale scores also tended to show improvement. Correlations between the change in the RiiQ™ Symptoms Scale scores and change in PGI-S and PGI-H were mostly moderate (*r* = 0.33 [URT] to 0.43 [Respiratory Symptoms] for PGI-S; *r* =  − 0.27 [LRT] to − 0.43 [Systemic Symptoms] for PGI-H) (Table 3). Because PGI-S and PGI-H scores document patient-reported change in the severity of their respiratory illness and overall health, respectively, these moderate associations, along with the linear trend seen in the pattern of mean change scores, demonstrate responsiveness to change.

**Table 3:** Ability to detect change of the RiiQ™ Symptoms Scale in vaccine trial

Global measure	Correlation, n RiiQ™ Symptoms Scale change score (from ARI PRO eDiary Day 1 to ARI Day 29)			
	Respiratory symptoms	LRT scale	URT scale	Systemic symptoms
Change in PGI-S	0.43, 733	0.37, 733	0.33, 733	0.39, 732
Change in PGI-H	− 0.37, 793	− 0.27, 793	− 0.37, 793	− 0.43, 790
PGI-C	0.22, 829	0.18, 829	0.17, 830	0.15, 829

The PGI-S was scored 0 (I feel fine) to 3 (I feel extremely ill) while the PGI-H was scored 0 (Very poor) to 4 (Very good). The PGI-C was scored − 3 (Much better) to 3 (Much worse). The PGI-H was scored 0 (Very poor) to 4 (Very good)

Pearson correlations were computed with the change in PGI-S and change in PGI-H; polyserial correlations were computed with the PGI-C

*hARI* acute respiratory infection, *LRT* lower respiratory tract, *PGI-C* Patient Global Impression of Change; PGI-H, Patient Global Impression of Health; PGI-S, Patient Global Impression of Severity; PRO, patient-reported outcome; RiiQ™, Respiratory Infection Intensity and Impact Questionnaire; URT, upper respiratory tract

## Discussion

This study used data from a vaccine trial and 2 observational studies (with samples of hospitalized patients) to evaluate the psychometric properties of the RiiQ™ symptoms scales for the purpose of assessing the efficacy of new RSV vaccines and treatments. Importantly, the vaccine trial and HARTI study populations were comprised primarily of older adults, including participants with risk factors for severe RSV disease, and thus representative of adults who may be vulnerable to serious RSV-mediated illness [10, 11]. The RiiQ™ items produced logical patterns of upper and lower respiratory symptoms across the study populations. Consistent with the varying contexts of use, participants at the early onset of an RSV infection from the vaccine study had fewer symptoms than participants hospitalized for ARI, and by the follow-up period, symptoms had resolved in almost all participants. In the observational studies, many symptoms resolved by follow-up, but some symptoms (e.g., moderate-to-severe shortness of breath and cough) remained in approximately 9.0–22.0% of patients.

Results of this evaluation provide support for the structure, reliability, validity, and responsiveness of the RiiQ™ Symptoms Scale summary scores (Respiratory [LRT and URT] and Systemic). Inter-item correlations, CFA results, and internal consistency reliability coefficients were generally positive and supported the proposed scoring. The two-factor CFA model hypothesized by Osborne and colleagues [7] fit the variance–covariance matrix of the respiratory-related symptom items and most Cronbach’s alpha values (internal consistency was well above the 0.70 threshold), although the LRT items (cough, wheezing, shortness of breath, expectoration) were more consistently related than the URT items (nasal congestion and sore throat). Overall, these results support the formation of Respiratory (LRT and URT) and Systemic scores, which can be viewed as cumulative indices. Moderate to strong correlations for LRT symptoms summary scores with the clinical questionnaire summary score and with the PGI-S supported construct validity and consistency between clinician and patient ratings. The correlations with URT symptoms summary scores were moderate among RSV-negative patients but small among RSV-positive patients. Finally, patterns of mean change and correlations between RiiQ™ Symptoms Scale summary scores and change in PGI-S and PGI-H scores provided evidence to support the responsiveness of the RiiQ™ Symptoms Scale summary scores.

Although established PRO measures exist for other respiratory illnesses (eg, fluiiQ™ [7] and Flu-PRO [22] for influenza), and other studies of RSV have made use of influenza-specific PROs [23–25], the RiiQ™ is a novel PRO that was specifically designed for assessing RSV symptoms. While the two pathogens can present with similar clinical symptoms, influenza-specific PROs may not fully capture the unique clinical symptomatology of RSV [4]. Further, the RiiQ™ is a short, simple PRO measure that can easily be administered in clinical settings [4], in contrast to prior influenza-specific PRO measures. Additional studies evaluating the usefulness of the RiiQ™ in clinical practice are needed.

Limitations of this study should be recognized. The vaccine trial was conducted in the US only, and the results may not be generalizable to other adult populations with RSV. The sample size for Observational Study 2 was small, with 20 RSV-positive patients enrolling and completing the study. In addition, because sample sizes for some analysis subgroups were small, some analyses were conducted with the full study sample. Further, analyses of internal consistency relied on Cronbach’s alpha, which may have underestimated reliability given the small sample sizes of RSV-positive patients, limited variability in the scores, and nonnormal distributions. The factor analyses were limited by both small sample sizes and low variability, and it was not feasible to fit a two-factor model for the Respiratory Symptoms and Systemic Symptoms scales, or to fit a bifactor model fitting these scales against an overall model of all items. Future evaluations of the RiiQ™ using different data sets should incorporate these factor analyses to gain additional insights into the structure of the RiiQ™.

## Conclusion

An extensive range of psychometric tests applied to the RiiQ™ symptoms summary scores provide evidence of reliability, construct validity, discriminating ability, and responsiveness of the RiiQ™ for use in clinical studies to assess the onset and severity of RSV symptoms. Taken together with the rigorous process used to develop the RiiQ™, the results of this study support the use of the RiiQ™ symptoms summary scores to evaluate efficacy in the context of RSV vaccine and treatment studies.

## Supplementary Information

**Additional file 1. **Summary tables of study samples, variables included, study populations, and psychometric properties of the RiiQ™.

## Data Availability

The data sharing policy of Janssen Pharmaceutical Companies of Johnson & Johnson is available at https://www.janssen.com/clinical-trials/transparency. As noted on this site, requests for access to the study data can be submitted through Yale Open Data Access (YODA) Project site at http://yoda.yale.edu.

## Abbreviations

ANOVA: Analysis of variance
ARI: Acute respiratory infection
CFA: Confirmatory factor analysis
CFI: Comparative fit index
COPD: Chronic obstructive pulmonary disease
FluiiQ™: Influenza Intensity and Impact Questionnaire
hMPV: Human metapneumovirus
IADL: Instrumental Activities of Daily Living
ICC: Intraclass correlation coefficient
LRT: Lower respiratory tract
PGI-C: Patient Global Impression of Change
PGI-H: Patient Global Impression of Health
PGI-S: Patient Global Impression of Severity
PRO: Patient-reported outcome
RiiQ™: Respiratory Infection Intensity and Impact Questionnaire
RMSEA: Root mean square error of approximation
RSV: Respiratory syncytial virus
RT-PCR: Reverse transcription polymerase chain reaction
TLI: Tucker–Lewis Index
URT: Upper respiratory tract
