# Supplementary material for: Evaluation of symptoms in respiratory syncytial virus infection in adults: psychometric evaluation of the Respiratory Infection Intensity and Impact Questionnaire™ symptom scores
Source: J Patient Rep Outcomes. 2023 Jun 1;7:51. doi: 10.1186/s41687-023-00593-9 (PMC10235291; doi:10.1186/s41687-023-00593-9)
Supplement: Supplementary file 1 — Additional file 1. Summary tables of study samples, variables included, study populations, and psychometric properties of the RiiQ™. [file 41687_2023_593_MOESM1_ESM.docx]

# Supplemental Material

Table S1. Summary of the RiiQ™ Time Points and Population Used in the Key Psychometric Evaluation Results

| Study | Population  (sample size) | Time points (sample size) | RiiQ™ item administration |
| --- | --- | --- | --- |
| Vaccine trial | RSV-positive:   - ARI event and confirmed RSV with no coinfections (n=60)   Not RSV-positive:   - ARI event and confirmed not RSV with no coinfections (n=1,615)   All ARI:   - ARI event with no coinfections (n=1,675) | First ARI event:   - ARI PRO eDiary Day 1 (first day with a RiiQ™ response on the PRO eDiary device) - ARI Day 3-5 (site eDevice) - ARI Day 29 (site eDevice) | RiiQ™ symptom-related and Impact on Daily Activity items at all time points (both devices)  RiiQ™ Impact on Emotions items and Impact on Relationship items at site visits |
| HARTI | RSV-positive:   - Confirmed RSV - With no coinfections (n=100) | - Baseline: 2 days (48 hours) after screening for ARI (interview) - Discharge: Closest completion within the 2 days prior to planned discharge (interview) - Follow-up: 1 month (phone interview) | RiiQ™ symptom-related items at all time points |
| Observational Study 2 | RSV-positive:   - Confirmed RSV (n=20) | - Baseline: Day after screening for ARI (interview) - Discharge: Closest completion within the 2 days prior to discharge (interview) - Follow-up: 10 ± 2 days after hospital discharge (telephone interview) | RiiQ™ symptom-related items at all time points |

ARI=acute respiratory infection; PRO=patient-reported outcome; RiiQ™=Respiratory Infection Intensity and Impact Questionnaire; RSV=respiratory syncytial virus.

Table S2. Other Study Measures Used in the Key Psychometric Evaluation of the RiiQ™

| Measure | Description | Response options | Study |
| --- | --- | --- | --- |
| PGI‑S | Rate the severity of their respiratory illness | 0=I feel fine (no respiratory illness)  1=I feel a little ill  2=I feel very ill  3=I feel extremely ill | Vaccine trial   - ARI PRO eDiary Day 1 - ARI Day 3-5 (Site)^a^ - ARI Day 29 (Site) |
| PGI‑H | Rate their overall impression of their health status today on the following scale | 0=Very poor  1=Poor  2=Fair  3=Good  4=Very good | Vaccine trial   - ARI PRO eDiary Day 1 - ARI Day 3-5 (Site)^a^ - ARI Day 29 (Site) |
| PGI‑C | Rate the amount of change in their health each day during an ARI episode | −3=Much better  −2=Somewhat better  −1=A little better  0=About the same/no change  1=A little worse  2=Somewhat worse  3=Much worse | Vaccine trial   - ARI PRO eDiary Day 1 - ARI Day 3-5 (Site)^a^ - ARI Day 29 (Site) |
| Clinical questionnaire  (physical examination) | Symptoms   - Cough - Sputum production - Shortness of breath - Malaise (tiredness)   Upper respiratory   - Nasal discharge - Pharyngitis - Sinus tenderness   Lower respiratory   - Dyspnea - Rales, rhonchi, or other - Wheezing - Respiratory effort^b^ | 0 (No symptoms) to 3 (Bothersome most of the time, interfering with other activities) | Vaccine trial   - ARI Day 3-5 (Site) - ARI Day 29 (Site)   HARTI   - Baseline - Discharge   Observational Study 2   - Baseline - Discharge |
| Responsiveness item | Overall since this time yesterday (for in-hospital interview) or since last interview (for the remote interview) are you: <response options> | - Much worse - Worse - The same - Better - Much better | HARTI   - Baseline - Discharge - Follow-up   Observational Study 2   - Baseline - Discharge - Follow-up |
| Lawton-Brody IADL Scale | Participants rated functioning in eight domains required for independent living: ability to use a telephone, shopping, food preparation, housekeeping, laundry, mode of transportation, responsibility for own medications, and ability to handle finances (Lawton and Brody, 1969) | 0 (low function, dependent) to 8 (high function, independent)  (scored irrespective of gender) | Vaccine trial   - ARI Day 3-5 (Site) - ARI Day 29 (Site)   Observational Study 2   - Baseline - Discharge - Follow-up   HARTI   - Baseline - Discharge - Follow-up |

ARI=acute respiratory infection; IADL=Instrumental Activities of Daily Living; PGI-C=Patient Global Impression of Change; PGI-H=Patient Global Impression of Health; PGI‑S=Patient Global Impression of Severity; PRO=patient-reported outcome; RiiQ™=Respiratory Infection Intensity and Impact Questionnaire.

^a^ Due to an electronic record transfer issue, PGI-S scores were not collected on ARI Day 3-5 for all participants.

^b^ The Respiratory effort item of the clinical questionnaire was not collected in HARTI.

Table S3. Patient Characteristics and Disease Status

A. Vaccine Trial

| Patient characteristic | ARI with no coinfections | | ARI with coinfections (n=329) | ARI with unknown coinfections^a^ (n=124) | Non-ARI (n=3,654) | Total sample (N=5,782) |
| --- | --- | --- | --- | --- | --- | --- |
|  | RSV (n=60) | Non-RSV (n=1,615) |  |  |  |  |
| Sex, n (%) | | | | | | |
| Male | 23 (38.3) | 659 (40.8) | 128 (38.9) | 59 (47.6) | 1,579 (43.2) | 2,448 (42.3) |
| Female | 37 (61.7) | 956 (59.2) | 201 (61.1) | 65 (52.4) | 2,075 (56.8) | 3,334 (57.7) |
| Age (years) | | | | | | |
| Mean (SD), median | 71.5 (5.1), 71.0 | 71.3 (5.1), 70.0 | 71.1 (5.3), 70.0 | 71.2 (4.8), 70.0 | 71.9 (5.5), 71.0 | 71.7 (5.4), 71.0 |
| Min, max | 65.0, 86.0 | 65.0, 91.0 | 65.0, 90.0 | 65.0, 86.0 | 65.0, 98.0 | 65.0, 98.0 |
| Race, n (%) | | | | | | |
| White | 60 (100.0) | 1,508 (93.7) | 312 (94.8) | 112 (91.1) | 3,356 (92.3) | 5,348 (92.9) |
| Black or African American | 0 (0.0) | 86 (5.3) | 9 (2.7) | 9 (7.3) | 213 (5.9) | 317 (5.5) |
| Asian | 0 (0.0) | 6 (0.4) | 2 (0.6) | 0 (0.0) | 24 (0.7) | 32 (0.6) |
| American Indian or Alaska Native | 0 (0.0) | 1 (0.1) | 4 (1.2) | 1 (0.8) | 11 (0.3) | 17 (0.3) |
| Native Hawaiian or other Pacific Islander | 0 (0.0) | 3 (0.2) | 0 (0.0) | 0 (0.0) | 18 (0.5) | 21 (0.4) |
| Multiple | 0 (0.0) | 5 (0.3) | 2 (0.6) | 1 (0.8) | 15 (0.4) | 23 (0.4) |
| Ethnicity, n (%) | | | | | | |
| Hispanic or Latino | 0 (0.0) | 43 (2.7) | 13 (4.0) | 4 (3.3) | 121 (3.3) | 181 (3.2) |
| Not Hispanic or Latino | 60 (100.0) | 1,556 (97.3) | 316 (96.0) | 117 (96.7) | 3,503 (96.7) | 5,552 (96.8) |
| Number of comorbidities of interest | | | | | | |
| Mean (SD), median | 1.9 (1.1), 2.0 | 2.0 (1.3), 2.0 | 1.8 (1.2), 2.0 | 2.2 (1.3), 2.0 | 1.9 (1.3), 2.0 | 1.9 (1.3), 2.0 |
| 25th percentile | 1.0 | 1.0 | 1.0 | 1.0 | 1.0 | 1.0 |
| 75th percentile | 2.5 | 3.0 | 2.0 | 3.0 | 3.0 | 3.0 |
| Min, max | 0.0, 5.0 | 0.0, 7.0 | 0.0, 5.0 | 0.0, 5.0 | 0.0, 8.0 | 0.0, 8.0 |
| Type of comorbidity, n (%) | | | | | | |
| COPD | 3 (5.0) | 126 (7.8) | 24 (7.3) | 12 (9.7) | 263 (7.2) | 428 (7.4) |
| CHF | 1 (1.7) | 37 (2.3) | 3 (0.9) | 3 (2.4) | 69 (1.9) | 113 (2.0) |
| Asthma | 6 (10.0) | 167 (10.3) | 30 (9.1) | 20 (16.1) | 309 (8.5) | 532 (9.2) |
| Other chronic heart disease | 4 (6.7) | 173 (10.7) | 29 (8.8) | 20 (16.1) | 445 (12.2) | 671 (11.6) |
| Other chronic lung disease | 1 (1.7) | 31 (1.9) | 9 (2.7) | 0 (0.0) | 45 (1.2) | 86 (1.5) |

ARI=acute respiratory infection; CHF=chronic heart failure; COPD=chronic obstructive pulmonary disease; RSV=respiratory syncytial virus; RT-PCR= reverse-transcription polymerase chain reaction; SD=standard deviation.

Notes: Coinfections are based on the GeneXpert and GenMark results. At the time of the primary analysis, GenMark results were available for only a part of the ARIs.

^a^ This sample is a combination of participants with nonstable RT-PCR swabs (e.g., the swab was not stored correctly) or participants with no RT-PCR result available during the ARI.

B. HARTI and Observational Study 2

| Patient characteristic | HARTI | | | | | Observational Study 2  (N=24) |
| --- | --- | --- | --- | --- | --- | --- |
|  | RSV-only infection (n=100) | Influenza-only infection (n=259) | hMPV-only infection (n=36) | RSV and influenza infection (n=2) | Influenza and hMPV-only infection (n=1) |  |
| Sex, n (%) |  |  |  |  |  |  |
| Male | 38 (38.0) | 113 (43.6) | 12 (33.3) | 1 (50.0) | 0 (0.0) | 10 (41.7) |
| Female | 62 (62.0) | 146 (56.4) | 24 (66.7) | 1 (50.0) | 1 (100.0) | 14 (58.3) |
| Age (years) |  |  |  |  |  |  |
| Mean (SD), median | 66.5 (17.9), 69.0 | 64.7 (15.3), 65.0 | 63.8 (14.2), 63.0 | 74.0 (1.4), 74.0 | 32.0 (-), 32.0 | 70.4 (13.9), 70.0 |
| Min, max | 18.0, 98.0 | 20.0, 96.0 | 28.0, 90.0 | 73.0, 75.0 | 32.0, 32.0 | 43.0, 92.0 |
| Hemisphere, n (%) |  |  |  |  |  |  |
| Northern | 75 (75.0) | 200 (77.2) | 30 (83.3) | 2 (100.0) | 0 (0.0) | 24 (100.0) |
| Southern | 25 (25.0) | 59 (22.8) | 6 (16.7) | 0 (0.0) | 1 (100.0) | 0 (0.0) |
| Country, n (%) |  |  |  |  |  |  |
| Belgium | - | - | - | - | - | 24 (100.0) |
| Argentina | 9 (9.0) | 21 (8.1) | 1 (2.8) | 0 (0.0) | 0 (0.0) | - |
| Australia | 8 (8.0) | 0 (0.0) | 4 (11.1) | 0 (0.0) | 0 (0.0) | - |
| Brazil | 4 (4.0) | 19 (7.3) | 0 (0.0) | 0 (0.0) | 0 (0.0) | - |
| Canada | 1 (1.0) | 0 (0.0) | 0 (0.0) | 0 (0.0) | 0 (0.0) | - |
| Germany | 3 (3.0) | 20 (7.7) | 1 (2.8) | 0 (0.0) | 0 (0.0) | - |
| France | 7 (7.0) | 12 (4.6) | 1 (2.8) | 0 (0.0) | 0 (0.0) | - |
| Japan | 8 (8.0) | 1 (0.4) | 4 (11.1) | 0 (0.0) | 0 (0.0) | - |
| Korea | 6 (6.0) | 28 (10.8) | 1 (2.8) | 0 (0.0) | 0 (0.0) | - |
| Mexico | 1 (1.0) | 2 (0.8) | 0 (0.0) | 0 (0.0) | 0 (0.0) | - |
| Malaysia | 6 (6.0) | 27 (10.4) | 0 (0.0) | 0 (0.0) | 0 (0.0) | - |
| US | 43 (43.0) | 110 (42.5) | 23 (63.9) | 2 (100.0) | 0 (0.0) | - |
| South Africa | 4 (4.0) | 19 (7.3) | 1 (2.8) | 0 (0.0) | 1 (100.0) | - |
| Number of comorbidities |  |  |  |  |  |  |
| Mean (SD), Median | 2.7 (2.0), 2.0 | 2.4 (1.8), 2.0 | 2.4 (1.6), 2.0 | 4.5 (0.7), 4.5 | 0.0 (-), 0.0 | 1.5 (1.1), 1.0 |
| 25th percentile | 1.0 | 1.0 | 1.0 | 4.0 | 0.0 | 1.0 |
| 75th percentile | 4.0 | 3.0 | 3.0 | 5.0 | 0.0 | 2.5 |
| Min, max | 0.0, 7.0 | 0.0, 8.0 | 0.0, 6.0 | 4.0, 5.0 | 0.0, 0.0 | 0.0, 3.0 |
| Type of comorbidity, n (%) |  |  |  |  |  |  |
| Asthma | 27 (27.0) | 34 (13.1) | 8 (22.2) | 0 (0.0) | 0 (0.0) | 0 (0.0) |
| Chronic heart disease | 32 (32.0) | 95 (36.7) | 11 (30.6) | 1 (50.0) | 0 (0.0) | 3 (12.5) |
| Chronic renal disease | 13 (13.0) | 35 (13.5) | 5 (13.9) | 2 (100.0) | 0 (0.0) | 5 (20.8) |
| COPD | 31 (31.0) | 56 (21.6) | 13 (36.1) | 1 (50.0) | 0 (0.0) | 4 (16.7) |

COPD=chronic obstructive pulmonary disease; hMPV=human metapneumovirus; RiiQ™=Respiratory Infection Intensity and Impact Questionnaire; RSV=respiratory syncytial virus; SD=standard deviation; US=United States.

Notes: The Observational Study 2 psychometric analysis sample consists of subjects in the full analysis set. The HARTI psychometric analysis sample consists of subjects in the substudy sample who had a completed RiiQ™ assessment at baseline.

****Table S4. Percentage of Participants With RSV-Positive Disease Status Reporting “None” for RiiQ™ Symptom Items at the Initial Assessment****

| RiiQ™ Symptom Scale and item content* | Percentage of “None” out of the RSV-positive sample at the initial assessment | | | |
| --- | --- | --- | --- | --- |
|  | Vaccine trial | | HARTI | Observational Study 2 |
|  | ARI Day 1 (PRO eDiary) (n=40-41) | ARI Day 3-5 (site visit) (n=49-52) | Baseline^†^ (n=100) | Baseline (n=20) |
| Respiratory Symptoms, % |  |  |  |  |
| LRT |  |  |  |  |
| a. Cough | 10.0 | 14.0 | 6.0 | 0.0 |
| j. Wheezing | 62.5 | 59.2 | 39.0 | 40.0 |
| k. Expectoration | 35.0 | 36.7 | 33.0 | 30.0 |
| l. Shortness of breath | 65.0 | 66.7 | 28.0 | 20.0 |
| URT |  |  |  |  |
| b. Sore throat | 30.0 | 54.9 | 74.0 | 80.0 |
| d. Nasal congestion | 2.4 | 5.9 | 33.0 | 55.0 |
| Systemic Symptoms, % |  |  |  |  |
| c. Headache | 47.5 | 49.0 | 64.0 | 70.0 |
| e. Fever | 67.5 | 71.2 | 66.0 | 75.0 |
| f. Body pains | 60.0 | 52.9 | 50.0 | 60.0 |
| g. Fatigue | 9.8 | 13.7 | 29.0 | 25.0 |
| h. Neck pain | 77.5 | 70.6 | 76.0 | 85.0 |
| i. Interrupted sleep | 37.5 | 42.0 | 34.0 | 35.0 |
| m. Appetite | 48.8 | 60.0 | 60.0 | 40.0 |

ARI=acute respiratory infection; LRT=lower respiratory tract; PRO=patient-reported outcome; RiiQ™=Respiratory Infection Intensity and Impact Questionnaire; RSV = respiratory syncytial virus; URT=upper respiratory tract.

Note: Orange text indicates a ceiling effect (i.e., > 50%).

* Items truncated. Complete items available from the author.

^†^ Initial (baseline) assessment was a maximum of 48 hours after the screening visit.

Table S5. Percentage of ****Participants With RSV-Positive Disease Status Reporting**** Moderate to Severe RiiQ™ Symptom Items at Select Timepoints: Sample=RSV-Positive

| RiiQ™ Symptom Scale and item content* | Percentage of “moderate to severe” among RSV-positive sample | | | | | | |
| --- | --- | --- | --- | --- | --- | --- | --- |
|  | Used as an initial assessment in the analyses | | | | Used as a final assessment in the analyses | | |
|  | Vaccine trial | | HARTI | Observational Study 2 | Vaccine trial | HARTI | Observational Study 2 |
|  | ARI Day 1 (PRO eDiary) (n=40-41) | ARI Day 3-5 (site visit) (n=49-52) | Baseline^†^ (n=100) | Baseline (n=20) | ARI Day 29 (n=53-55) | Follow-up (n=86) | Follow-up (n=18) |
| Respiratory Symptoms, % |  |  |  |  |  |  |  |
| LRT |  |  |  |  |  |  |  |
| a. Cough | 60.0 | 46.0 | 55.0 | 70.0 | 0.0 | 9.3 | 16.7 |
| j. Wheezing | 10.0 | 16.3 | 38.0 | 30.0 | 0.0 | 7.0 | 0.0 |
| k. Expectoration | 27.5 | 24.5 | 33.0 | 30.0 | 0.0 | 5.8 | 11.1 |
| l. Shortness of breath | 7.5 | 5.9 | 49.0 | 50.0 | 1.9 | 19.8 | 22.2 |
| URT |  |  |  |  |  |  |  |
| b. Sore throat | 27.5 | 19.6 | 8.0 | 5.0 | 0.0 | 1.2 | 0.0 |
| d. Nasal congestion | 56.1 | 58.8 | 27.0 | 30.0 | 0.0 | 11.6 | 0.0 |
| Systemic Symptoms, % |  |  |  |  |  |  |  |
| c. Headache | 17.5 | 18.4 | 11.0 | 5.0 | 0.0 | 9.3 | 0.0 |
| e. Fever | 12.5 | 5.8 | 4.0 | 15.0 | 0.0 | 0.0 | 0.0 |
| f. Body pains | 12.5 | 13.7 | 24.0 | 25.0 | 0.0 | 12.8 | 5.6 |
| g. Fatigue | 31.7 | 31.4 | 42.0 | 40.0 | 3.7 | 21.0 | 22.2 |
| h. Neck pain | 10.0 | 5.9 | 10.0 | 5.0 | 0.0 | 8.1 | 0.0 |
| i. Interrupted sleep | 32.5 | 30.0 | 43.0 | 40.0 | 0.0 | 15.1 | 11.1 |
| m. Appetite | 12.2 | 14.0 | 18.0 | 25.0 | 1.8 | 5.8 | 0.0 |

ARI=acute respiratory infection; LRT=lower respiratory tract; PRO=patient-reported outcome; RiiQ™=Respiratory Infection Intensity and Impact Questionnaire; RSV=respiratory syncytial virus; URT=upper respiratory tract.

* Items truncated. Complete items available from the author.

^†^ Initial (baseline) assessment was a maximum of 48 hours after the screening visit.

Table S6. ****Lower and Upper Respiratory Symptoms Summary Scores Among Participants With RSV-Positive Disease Status****

| Study/timepoint/score | n | Mean (SD) | Median | Min, max | Highest (severe)/ lowest (none) score (%) |
| --- | --- | --- | --- | --- | --- |
| **Vaccine trial** |  |  |  |  |  |
| ARI Day 1 |  |  |  |  |  |
| Lower Respiratory Tract Symptom summary score | 40 | 0.9 (0.6) | 0.8 | 0.0, 2.8 | 0.0/7.5 |
| Upper Respiratory Tract Symptom summary | 41 | 1.4 (0.6) | 1.5 | 0.5, 3.0 | 2.4/0.0 |
| ARI Day 3-5 |  |  |  |  |  |
| Lower Respiratory Tract Symptom summary | 52 | 0.8 (0.6) | 0.8 | 0.0, 2.5 | 0.0/9.6 |
| Upper Respiratory Tract Symptom summary | 53 | 1.2 (0.6) | 1.0 | 0.0, 2.5 | 0.0/3.8 |
| ARI Day 29 |  |  |  |  |  |
| Lower Respiratory Tract Symptom summary | 54 | 0.1 (0.2) | 0.0 | 0.0, 1.3 | 0.0/75.9 |
| Upper Respiratory Tract Symptom summary | 55 | 0.1 (0.2) | 0.0 | 0.0, 0.5 | 0.0/87.3 |
| HARTI |  |  |  |  |  |
| Baseline^*^ |  |  |  |  |  |
| Lower Respiratory Tract Symptom summary | 100 | 1.3 (0.8) | 1.3 | 0.0, 3.0 | 3.0/3.0 |
| Upper Respiratory Tract Symptom summary | 100 | 0.7 (0.7) | 0.5 | 0.0, 3.0 | 1.0/28.0 |
| 1-month follow-up |  |  |  |  |  |
| Lower Respiratory Tract Symptom summary | 86 | 0.5 (0.5) | 0.3 | 0.0, 2.3 | 0.0/39.5 |
| Upper Respiratory Tract Symptom summary | 86 | 0.3 (0.4) | 0.0 | 0.0, 2.5 | 0.0/67.4 |

Note: Scale scores reported on a 0-3 scale with higher scores indicative of greater severity.

ARI=acute respiratory infection; RSV=respiratory syncytial virus; SD=standard deviation.

^*^ Initial (baseline) assessment was a maximum of 48 hours after the screening visit.

Table S7. Test-Retest Reliability of the RiiQ™ Symptoms Scales

| RiiQ™ Symptoms Scale | ICC (95% CI, n) | |
| --- | --- | --- |
|  | Observational Study 2 (RSV-positive) identical responsiveness item response  on 2 consecutive days | Vaccine trial (RSV-positive) identical PGI-S response  on 2 consecutive days |
| Respiratory Symptoms | 0.77 (0.09 to 0.95), 8 | 0.83 (0.73 to 0.90), 54 |
| LRT | 0.86 (0.43 to 0.97), 8 | 0.86 (0.78 to 0.92), 54 |
| URT | 0.41 (−0.18 to 0.83), 8 | 0.80 (0.67 to 0.88), 54 |
| Systemic Symptoms | 0.71 (0.04 to 0.94), 8 | 0.84 (0.74 to 0.90), 54 |

CI=confidence interval; ICC=intraclass correlation coefficients; LRT=lower respiratory tract; PGI-S=Patient Global Impression of Severity; RiiQ™=Respiratory Infection Intensity and Impact Questionnaire; RSV=respiratory syncytial virus; URT=upper respiratory tract.

Note: The Impact on Daily Activities items were only administered in the vaccine trial.

Table S8. Polychoric Correlations Between Clinician and Patient Ratings of Similar Constructs

| Clinician and patient ratings of symptom | Study | Timepoint | Polychoric correlation |
| --- | --- | --- | --- |
| Cough | Vaccine trial | ARI Day 3-5 | 0.79 |
|  |  | ARI Day 29 | 0.91 |
|  | HARTI | Baseline | 0.69 |
|  |  | Prior to Discharge | 0.81 |
|  | Observational Study 2 | Baseline | 0.83 |
|  |  | Prior to Discharge | 1.00 |
| Expectoration | Vaccine trial | ARI Day 3-5 | 0.68 |
|  |  | ARI Day 29 | 1.00 |
|  | HARTI | Baseline | 0.64 |
|  |  | Prior to Discharge | 0.73 |
|  | Observational Study 2 | Baseline | 0.07 |
|  |  | Prior to Discharge | 1.00 |
| Shortness of breath | Vaccine trial | ARI Day 3-5 | 0.92 |
|  |  | ARI Day 29 | 0.88 |
|  | HARTI | Baseline | 0.73 |
|  |  | Prior to Discharge | 0.91 |
|  | Observational Study 2 | Baseline | 0.76 |
|  |  | Prior to Discharge | 0.79 |
| Fatigue | Vaccine trial | ARI Day 3-5 | 0.66 |
|  |  | ARI Day 29 | 0.88 |
|  | HARTI | Baseline | 0.72 |
|  |  | Prior to Discharge | 0.61 |
|  | Observational Study 2 | Baseline | 0.93 |
|  |  | Prior to Discharge | 1.00 |
| Nasal congestion | Vaccine trial | ARI Day 3-5 | -0.02 |
|  |  | ARI Day 29 | 0.58 |
|  | HARTI | Baseline | 0.29 |
|  |  | Prior to Discharge | 0.69 |
|  | Observational Study 2 | Baseline | 0.46 |
|  |  | Prior to Discharge | 1.00 |
| Sore throat | Vaccine trial | ARI Day 3-5 | 0.72 |
|  |  | ARI Day 29 | − |
|  | HARTI | Baseline | 0.68 |
|  |  | Prior to Discharge | 0.87 |
|  | Observational Study 2 | Baseline | 0.46 |
|  |  | Prior to Discharge | − |
| Shortness of breath  (episodes) | Vaccine trial | ARI Day 3-5 | 0.74 |
|  |  | ARI Day 29 | 1.00 |
|  | HARTI | Baseline | 0.69 |
|  |  | Prior to Discharge | 0.79 |
|  | Observational Study 2 | Baseline | 0.78 |
|  |  | Prior to Discharge | 0.63 |
| Wheezing  (expiration) | Vaccine trial | ARI Day 3-5 | 0.74 |
|  |  | ARI Day 29 | − |
|  | HARTI | Baseline | 0.75 |
|  |  | Prior to Discharge | 0.76 |
|  | Observational Study 2 | Baseline | 0.67 |
|  |  | Prior to Discharge | 0.53 |

"−"=All (or nearly all) responses were "none" on both the clinician-reported and patient-reported outcome measure; ARI=acute respiratory infection.

Table S9. Construct Validity Correlations of the RiiQ™ Symptoms Scale With the Clinician Questionnaire in Participants With RSV-Positive Disease Status

| Study (sample)/ timepoint/ clinician questionnaire | Pearson correlation between the RiiQ™ Symptoms Scale scores with clinician questionnaire | | | |
| --- | --- | --- | --- | --- |
|  | Respiratory Symptoms | LRT | URT | Systemic Symptoms |
| Vaccine trial (RSV-positive) |  |  |  |  |
| ARI Day 3-5 (site visit) (n=52 to 53) |  |  |  |  |
| Symptoms (cough, sputum production, shortness of breath, malaise) | 0.67*  (0.48, 0.79) | 0.66*  (0.46, 0.79) | 0.40*  (0.14, 0.60) | 0.47*  (0.23, 0.66) |
| Upper respiratory (nasal discharge, pharyngitis, sinus tenderness) | 0.13  (−0.15, 0.39) | 0.10  (−0.18, 0.36) | 0.14  (−0.14, 0.39) | 0.31*  (0.04, 0.54) |
| Lower respiratory (dyspnea, rales/rhonchi, wheezing, respiratory effort) | 0.44*  (0.19, 0.64) | 0.46*  (0.21, 0.65) | 0.25  (−0.02, 0.49) | 0.29*  (0.02, 0.52) |
| ARI Day 29 (site visit) (n=48 to 49) |  |  |  |  |
| Symptoms (cough, sputum production, shortness of breath, malaise) | 0.70*  (0.52, 0.82) | 0.80*  (0.67, 0.88) | 0.11  (−0.18, 0.38) | 0.27  (−0.01, 0.52) |
| Upper respiratory (nasal discharge, pharyngitis, sinus tenderness) | 0.31*  (0.02, 0.54) | 0.28  (−0.01, 0.52) | 0.26  (−0.03, 0.50) | 0.23  (−0.06, 0.48) |
| Lower respiratory (dyspnea, rales/rhonchi, wheezing, respiratory effort) | 0.52*  (0.27, 0.70) | 0.64*  (0.43, 0.78) | −0.06  (−0.33, 0.23) | 0.20  (−0.09, 0.46) |
| HARTI (RSV-positive, n=99) |  |  |  |  |
| Baseline^†^ |  |  |  |  |
| Symptoms (cough, sputum production, shortness of breath, malaise) | 0.64*  (0.50, 0.74) | 0.68*  (0.56, 0.77) | 0.31*  (0.12, 0.48) | 0.59*  (0.44, 0.70) |
| Upper respiratory (nasal discharge, pharyngitis, sinus tenderness) | 0.40*  (0.22, 0.58) | 0.32*  (0.13, 0.51) | 0.43*  (0.26, 0.60) | 0.37*  (0.18, 0.55) |
| Lower respiratory (dyspnea, rales/rhonchi, wheezing, respiratory effort) | 0.59*  (0.45, 0.72) | 0.64*  (0.52, 0.76) | 0.24*  (0.05, 0.43) | 0.41*  (0.25, 0.58) |
| Observational Study 2 (RSV-positive, n=20) |  |  |  |  |
| Baseline |  |  |  |  |
| Symptoms (cough, sputum production, shortness of breath, malaise) | 0.57*  (0.16, 0.81) | 0.51*  (0.07, 0.77) | 0.54*  (0.11, 0.79) | 0.70*  (0.36, 0.87) |
| Upper respiratory (nasal discharge, pharyngitis, sinus tenderness) | 0.20  (−0.25, 0.66) | 0.09  (−0.39, 0.56) | 0.41  (0.03, 0.79) | 0.17  (−0.28, 0.62) |
| Lower respiratory (dyspnea, rales/rhonchi, wheezing, respiratory effort) | 0.85*  (0.72, 0.98) | 0.87*  (0.76, 0.98) | 0.49*  (0.15, 0.83) | 0.52*  (0.19, 0.85) |

ARI=acute respiratory infection; LRT=lower respiratory tract; RiiQ™=Respiratory Infection Intensity and Impact Questionnaire; RSV=respiratory syncytial virus; URT=upper respiratory tract.

* *P*<0.05. Note: Cohen’s (1992) guideline for interpreting correlation coefficients was used to classify each magnitude: absolute values of correlations of 0.50 or greater are considered strong (blue), correlations that fall between 0.30 and 0.49 are moderate (grey), and those that fall between 0.10 and 0.29 are weak (no color).

^†^ Initial (baseline) assessment was a maximum of 48 hours after the screening visit.

Table S10. Construct Validity of the RiiQ™ Symptoms Scale With PGI-H and PGI‑S: Vaccine Trial

| Study (sample)/ timepoint/ global measure^a^ | Polyserial correlation between the RiiQ™ Symptoms Scale scores with global items, n | | | |
| --- | --- | --- | --- | --- |
|  | Respiratory Symptoms | LRT | URT | Systemic Symptoms |
| **Vaccine trial (RSV-positive)** |  |  |  |  |
| ARI Day 1 (PRO eDiary, n=33) |  |  |  |  |
| PGI-H | −0.39 | −0.40 | −0.18 | −0.51 |
| PGI-S | 0.54 | 0.58 | 0.19 | 0.36 |
| ARI Day 29 (site visit, n=48-54) |  |  |  |  |
| PGI-H | −0.71 | −0.73 | −0.40 | −0.63 |
| PGI-S | 0.75 | 0.79 | 0.33 | 0.47 |
| **Vaccine trial (all ARI)** |  |  |  |  |
| ARI Day 1 (PRO eDiary, n=1,059) |  |  |  |  |
| PGI-H | −0.48 | −0.38 | −0.42 | −0.59 |
| PGI-S | 0.55 | 0.46 | 0.44 | 0.50 |
| ARI Day 3-5 (site visit, n=135-183) |  |  |  |  |
| PGI-H | −0.38 | −0.32 | −0.35 | −0.41 |
| PGI-S | 0.44 | 0.41 | 0.34 | 0.20 |
| ARI Day 29 (site visit, n=1,133-1,231) |  |  |  |  |
| PGI-H | −0.55 | −0.50 | −0.45 | −0.48 |
| PGI-S | 0.65 | 0.60 | 0.53 | 0.48 |

ARI=acute respiratory infection; LRT=lower respiratory tract; PGI-H=Patient Global Impression of Health; PGI‑S=Patient Global Impression of Severity; PRO=patient-reported outcome; RiiQ™=Respiratory Infection Intensity and Impact Questionnaire; RSV=respiratory syncytial virus; URT=upper respiratory tract.

Notes: The sample size for RSV-positive at ARI Day 3-5 (Site) for the global items was n<5 (due to an electronic record transfer issue). Cohen’s (1992) guideline for interpreting correlation coefficients was used to classify each magnitude: absolute values of correlations of 0.50 or greater are considered strong (blue), correlations that fall between 0.30 and 0.49 are moderate (grey), and those that fall between 0.10 and 0.29 are weak (no color).

^a^ The PGI-S was scored 0 (I feel fine) to 3 (I feel extremely ill) while the PGI-H was scored 0 (Very poor) to 4 (Very good).

Table S11. Construct Validity of the RiiQ™ Symptoms Scale With Lawton-Brody IADL Total Score

| Sample/  study/  timepoint | Pearson correlation between the RiiQ™ Symptoms Scale scores with Lawton-Brody IADL total score | | | |
| --- | --- | --- | --- | --- |
|  | Respiratory Symptoms | LRT | URT | Systemic  Symptoms |
| **RSV-positive** |  |  |  |  |
| Vaccine trial |  |  |  |  |
| ARI Day 3-5 (site visit, n=51-52) | −0.22 | −0.27 | −0.09 | −0.24 |
| HARTI |  |  |  |  |
| Baseline (n=98) | −0.06 | −0.15 | 0.15 | −0.13 |
| Observational Study 2 |  |  |  |  |
| Baseline (n=9) | 0.04 | 0.26 | −0.59 | −0.39 |

ARI=acute respiratory infection; IADL=Instrumental Activities of Daily Living; LRT=lower respiratory tract; RiiQ™=Respiratory Infection Intensity and Impact Questionnaire; RSV=respiratory syncytial virus; URT=upper respiratory tract.

* *P*<0.05.

Table S12. Known-Groups Validity of the RiiQ™ Symptoms Scale Scores by Asthma/COPD Status: Study 1 (Baseline) or Vaccine Trial (ARI Day 3-5)

| RiiQ™ Symptoms Scale/study | Sample | Asthma/COPD | No  asthma/COPD | ANOVA results |
| --- | --- | --- | --- | --- |
|  |  | Mean (SD), n | Mean (SD), n |  |
| Respiratory Symptoms | | | | |
| Vaccine trial | All ARI | 0.9 (0.6), 228 | 0.7 (0.5), 1,233 | *P*<0.0001 |
|  | RSV-positive | 1.1 (0.7), 8 | 0.9 (0.5), 45 | *P*=0.4357 |
| HARTI | All ARI | 1.3 (0.6), 145 | 0.9 (0.6), 253 | *P*<0.0001 |
|  | RSV-positive | 1.4 (0.6), 46 | 0.8 (0.5), 54 | *P*<0.0001 |
| LRT | | | | |
| Vaccine trial | All ARI | 0.9 (0.7), 227 | 0.6 (0.5), 1,232 | *P*<0.0001 |
|  | RSV-positive | 1.3 (0.8), 7 | 0.8 (0.6), 45 | *P*=0.0496 |
| HARTI | All ARI | 1.6 (0.7), 145 | 1.1 (0.7), 253 | *P*<0.0001 |
|  | RSV-positive | 1.7 (0.7), 46 | 1.0 (0.6), 54 | *P*<0.0001 |
| URT | | | | |
| Vaccine trial | All ARI | 1.0 (0.7), 234 | 1.0 (0.6), 1,246 | *P*=0.7199 |
|  | RSV-positive | 0.9 (0.9), 8 | 1.2 (0.6), 45 | *P*=0.2538 |
| HARTI | All ARI | 0.8 (0.8), 145 | 0.6 (0.7), 253 | *P*=0.0256 |
|  | RSV-positive | 0.9 (0.8), 46 | 0.6 (0.6), 54 | *P*=0.0156 |
| Systemic Symptoms | | | | |
| Vaccine trial | All ARI | 0.7 (0.6), 225 | 0.6 (0.5), 1,225 | *P*=0.0103 |
|  | RSV-positive | 0.6 (0.7), 7 | 0.7 (0.5), 45 | *P*=0.6977 |
| HARTI | All ARI | 0.9 (0.6), 145 | 0.8 (0.7), 253 | *P*=0.0135 |
|  | RSV-positive | 1.0 (0.6), 46 | 0.6 (0.5), 54 | *P*=0.0021 |

ANOVA=analysis of variance; ARI=acute respiratory infection; COPD=chronic obstructive pulmonary disease; LRT=lower respiratory tract; RiiQ™=Respiratory Infection Intensity and Impact Questionnaire; RSV=respiratory syncytial virus; SD=standard deviation; URT=upper respiratory tract.

Table S13. Known-Groups Validity of the RiiQ™ Symptoms Scale by PGI-S: Vaccine Trial (ARI PRO eDiary Day 1)

| RiiQ™ Symptoms Scale/sample | **PGI-S** | | | | **ANOVA results** |
| --- | --- | --- | --- | --- | --- |
|  | **I feel  fine** | **I feel  a little bit ill** | **I feel  very ill** | **I feel  extremely ill** |  |
|  | **Mean (SD), n** | **Mean (SD), n** | **Mean (SD), n** | **Mean (SD), n** |  |
| Respiratory Symptoms | | | | | |
| All ARI | 0.5 (0.3), 106 | 0.8 (0.4), 811 | 1.3 (0.5), 137 | *1.4 (0.6), 5* | Pairwise *P*<0.05, except I feel very ill vs. I feel extremely ill |
| RSV-positive | *0.7 (0.2), 2* | 0.9 (0.3), 21 | 1.3 (0.6), 10 | *-, 0* | I feel a little bit ill vs. I feel very ill (*P*=0.0256) |
| **LRT** |  |  |  |  |  |
| All ARI | 0.3 (0.3), 106 | 0.6 (0.5), 811 | 1.2 (0.6), 137 | *1.1 (0.8), 5* | Pairwise *P*<0.05, except I feel a little bit ill vs. I feel extremely ill & I feel very ill vs. I feel extremely ill |
| RSV-positive | *0.4 (0.2), 2* | 0.7 (0.4), 21 | 1.3 (0.7), 10 | *-, 0* | I feel a little bit ill vs. I feel very ill (*P*=0.0149) |
| **URT** |  |  |  |  |  |
| All ARI | 0.7 (0.5), 106 | 1.1 (0.6), 811 | 1.6 (0.6), 137 | *2.1 (0.7), 5* | Pairwise *P*<0.05, except I feel very ill vs. I feel extremely ill |
| RSV-positive | *1.3 (0.4), 2* | 1.3 (0.5), 21 | 1.5 (0.5), 10 | *-, 0* | No pairwise significant |
| Systemic Symptoms | | | | | |
| All ARI | 0.4 (0.4), 106 | 0.6 (0.5), 811 | 1.2 (0.5), 137 | *1.9 (0.2), 5* | Pairwise *P*<0.05 |
| RSV-positive | *0.4 (0.2), 2* | 0.6 (0.5), 21 | 0.9 (0.5), 10 | *-, 0* | No pairwise significant |

“-”=not computed

ANOVA=analysis of variance; ARI=acute respiratory infection; LRT=lower respiratory tract; PGI‑S=Patient Global Impression of Severity; PRO=patient-reported outcome; RiiQ™=Respiratory Infection Intensity and Impact Questionnaire; RSV=respiratory syncytial virus; SD=standard deviation; URT=upper respiratory tract.

Note: ANOVA test not computed with subgroups n ≤ 5 (indicated by *gray italicized text*).
The sample size was limited at Day 3-5 (Site) for the global items due to an electronic record transfer issue.

Table S14. Descriptive Statistics of Change in RiiQ™ Symptoms Scale by Change in PGI-S, Change in PGI-H, PGI-C: Vaccine Trial (Sample=All ARI)

| **Change score** | **Change score (from ARI PRO eDiary Day 1 to ARI Day 29) mean (SD), median, n^b^** | | | |
| --- | --- | --- | --- | --- |
|  | **Respiratory Symptoms** | **LRT scale** | **URT scale** | **Systemic**  **Symptoms** |
| **Change in PGI-S**^a^ |  |  |  |  |
| −3 (3-point improvement) | *−1.8 (0.1), −1.8, 2* | *−1.4 (0.2), −1.4, 2* | *−2.5 (0.7), −2.5, 2* | *−2.0 (0.2), −2.0, 2* |
| −2 (2-point improvement) | −1.2 (0.5), −1.2, 84 | −1.0 (0.6), −1.0, 84 | −1.4 (0.6), −1.5, 84 | −1.1 (0.6), −1.1, 84 |
| −1 (1-point improvement) | −0.7 (0.4), −0.7, 537 | −0.6 (0.5), −0.5, 537 | −1.1 (0.6), −1.0, 537 | −0.6 (0.4), −0.4, 536 |
| 0 (No change) | −0.4 (0.4), −0.5, 107 | −0.3 (0.4), −0.3, 107 | −0.7 (0.5), −0.5, 107 | −0.4 (0.5), −0.1, 107 |
| 1 (1-point worsening) | −0.1 (0.6), −0.3, 3 | 0.2 (0.9), −0.3, 3 | −0.5 (0.5), −0.5, 3 | −0.3 (0.1), −0.4, 3 |
| ≥ 2 (2-point or more worsening) | -, 0 | -, 0 | -, 0 | -, 0 |
| **Change in PGI-H**^a^ |  |  |  |  |
| ≤ −2 (2-point or more worsening) | -, 0 | -, 0 | -, 0 | -, 0 |
| −1 (1-point worsening) | −0.4 (0.5), −0.3, 11 | −0.3 (0.6), 0.0, 11 | −0.6 (0.6), −0.5, 11 | −0.3 (0.7), −0.4, 10 |
| 0 (No change) | −0.5 (0.4), −0.5, 134 | −0.4 (0.4), −0.3, 134 | −0.8 (0.6), −0.5, 134 | −0.3 (0.4), −0.3, 134 |
| 1 (1-point improvement) | −0.7 (0.4), −0.7, 332 | −0.5 (0.5), −0.5, 332 | −1.0 (0.5), −1.0, 332 | −0.5 (0.4), −0.4, 332 |
| 2 (2-point improvement) | −0.8 (0.4), −0.8, 246 | −0.7 (0.5), −0.5, 246 | −1.2 (0.6), −1.0, 246 | −0.7 (0.5), −0.7, 244 |
| 3 (3-point improvement) | −1.1 (0.5), −1.0, 66 | −0.8 (0.6), −0.8, 66 | −1.5 (0.6), −1.5, 66 | −1.1 (0.6), −1.0, 66 |
| 4 (4-point improvement) | −1.5 (0.4), −1.5, 4 | −1.4 (0.5), −1.3, 4 | −1.9 (0.3), −2.0, 4 | −2.0 (0.4), −2.1, 4 |
| **PGI-C** |  |  |  |  |
| Very much better | −0.8 (0.5), −0.7, 496 | −0.6 (0.5), −0.5, 496 | −1.1 (0.6), −1.0, 496 | −0.6 (0.5), −0.6, 496 |
| Much better | −0.8 (0.5), −0.8, 37 | −0.6 (0.6), −0.5, 37 | −1.0 (0.5), −1.0, 37 | −0.6 (0.5), −0.4, 37 |
| A little better | −0.6 (0.5), −0.7, 15 | −0.4 (0.6), −0.5, 15 | −0.8 (0.7), −1.0, 15 | −0.5 (0.5), −0.4, 15 |
| About the same | −0.7 (0.4), −0.7, 248 | −0.5 (0.5), −0.5, 248 | −1.0 (0.6), −1.0, 249 | −0.5 (0.5), −0.4, 248 |
| A little worse | −0.5 (0.6), −0.3, 25 | −0.3 (0.7), −0.3, 25 | −0.7 (0.7), −1.0, 25 | −0.6 (0.6), −0.4, 25 |
| Much worse | −0.6 (0.8), −0.4, 6 | −0.5 (1.0), −0.1, 6 | −0.8 (0.7), −1.0, 6 | −0.3 (0.7), 0.0, 6 |
| Very much worse | *−0.8 (0.6), −0.8, 2* | *−0.6 (0.2), −0.6, 2* | *−1.0 (1.4), −1.0, 2* | *−0.7 (0.6), −0.7, 2* |

ARI=acute respiratory infection; LRT=lower respiratory tract; PGI-C=Patient Global Impression of Change; PGI-H=Patient Global Impression of Health; PGI‑S=Patient Global Impression of Severity; PRO=patient-reported outcome; RiiQ™=Respiratory Infection Intensity and Impact Questionnaire, Version 2; SD=standard deviation; URT=upper respiratory tract.

Note: *Gray italicized text* indicates n<5. The sample size was limited at Day 3-5 (Site) for the global items due to an electronic record transfer issue.

^a^ The PGI-S was scored 0 (I feel fine) to 3 (I feel extremely ill) while the PGI-H was scored 0 (Very poor) to 4 (Very good). The PGI-C was scored -3 (Much better) to 3 (Much worse).

Figure S1. Box Plots of Known-Groups ANOVAs by PGI-H: Vaccine Trial, ARI With No Coinfections Sample (at ARI Day 3-5)


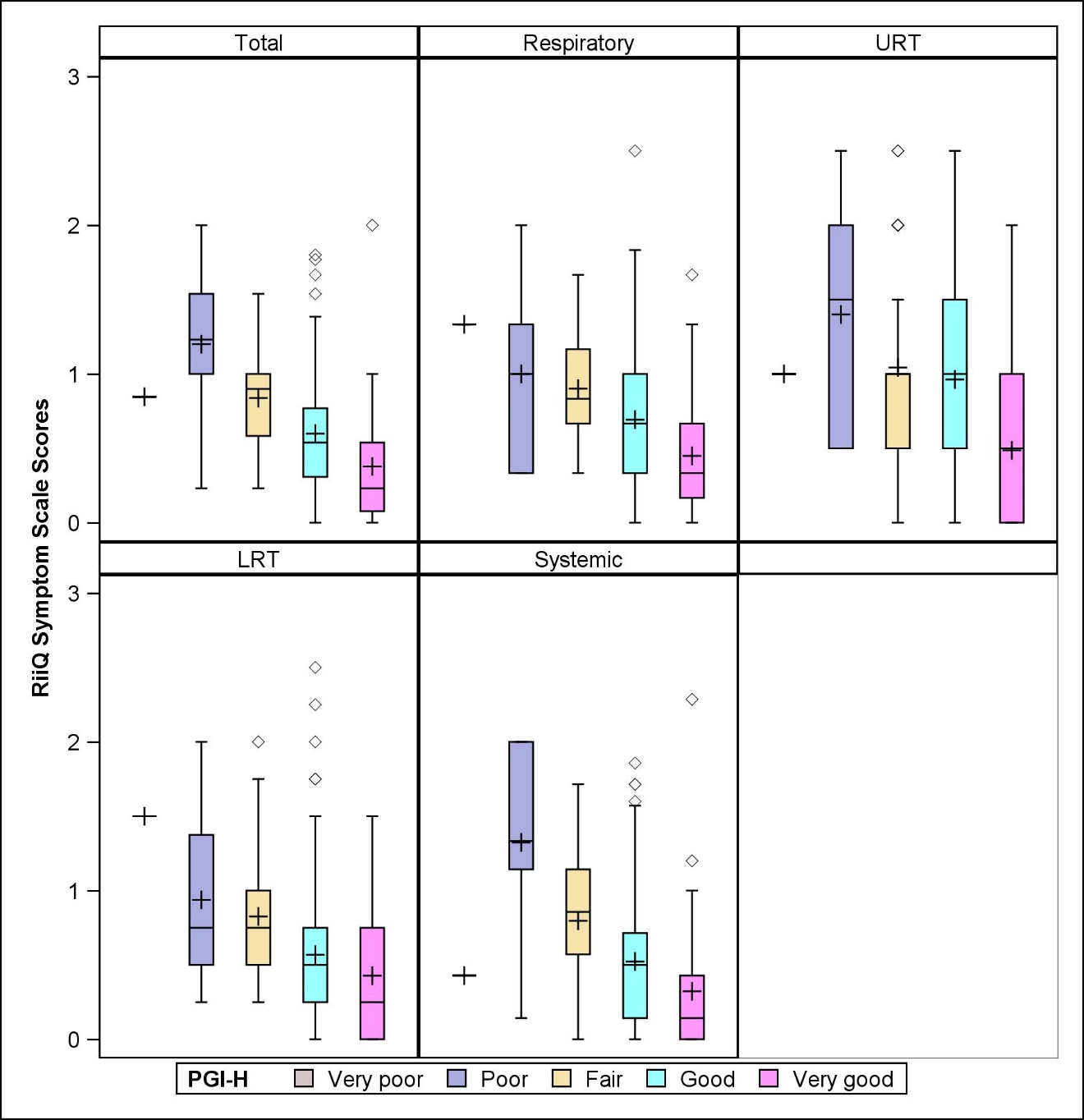


ANOVA=analysis of variance; ARI=acute respiratory infection; LRT=lower respiratory tract; PGI-H=Patient Global Impression of Health; URT=upper respiratory tract.

Note: The + symbol denotes the mean, and the horizontal lines inside the box denote the median. The diamond symbols represent values beyond the bounds of the whiskers.
